# A forensic case of hydranencephaly in a preterm neonate fully documented by postmortem imaging techniques

**DOI:** 10.1093/fsr/owad002

**Published:** 2023-01-13

**Authors:** Coraline Egger, Fabrice Dédouit, Bettina Schrag, Sylviane Hanquinet, Tony Fracasso

**Affiliations:** University Center of Legal Medicine Lausanne – Geneva (CURML), Unit of Forensic Medicine and Imaging, Rue Michel-Servet 1, 1211 Geneva, Switzerland; Service of Legal Medicine, Hospital of Rangueil, Avenue du Professeur Jean Poulhès 1, 31400 Toulouse, France; Service of Legal Medicine, Central Institute of the Hospital of Valais, Avenue du Grand-Champsec 86, 1951 Sion, Switzerland; Department of Pediatric Radiology, Children’s University Hospital of Geneva, Rue Gabrielle-Perret-Gentil 4, 1205 Geneva, Switzerland; University Center of Legal Medicine Lausanne – Geneva (CURML), Unit of Forensic Medicine and Imaging, Rue Michel-Servet 1, 1211 Geneva, Switzerland; University Center of Legal Medicine Lausanne – Geneva (CURML), Unit of Forensic Medicine and Imaging, Ch. de la Vulliette 4, 1000 Lausanne, Switzerland

**Keywords:** forensic imaging, postmortem magnetic resonance imaging, postmortem computed tomography, postmortem angiography, hydranencephaly

## Abstract

The authors present a medico-legal autopsy case of hydranencephaly in a male preterm newborn, fully documented by postmortem unenhanced and enhanced imaging techniques (postmortem computed tomography and postmortem magnetic resonance imaging). Hydranencephaly is a congenital anomaly of the central nervous system, consisting in almost complete absence of the cerebral hemispheres and replacement of the cerebral parenchyma by cerebrospinal fluid, rarely encountered in forensic medical practice. A premature baby was born during the supposed 22nd and 24th week of pregnancy in the context of a denial of pregnancy without any follow-up. The newborn died a few hours after birth and medico-legal investigations were requested to determine the cause of death and exclude the intervention of a third person in the lethal process. The external examination revealed neither traumatic nor malformative lesions. Postmortem imaging investigations were typical of hydranencephaly, and conventional medico-legal autopsy, neuropathological examination, and histological examination confirmed a massive necrotic-haemorrhagic hydranencephaly. This case represents in itself an association of out-of-the-ordinary elements making it worthy of interest.

**Key Points:**

- Postmortem unenhanced and enhanced imaging techniques (computed tomography and magnetic resonance imaging) were performed as complementary examination to conventional medico-legal investigations.
- Postmortem angiography of a preterm newborn is possible with catheterization of the umbilical blood vessels.
- Hydranencephaly is a congenital anomaly of the central nervous system, consisting in almost complete absence of the cerebral hemispheres and replacement of the brain by cerebrospinal fluid, for which several aetiologies have been postulated.

## Introduction

Hydranencephaly is an essentially foetal pathology, consisting in a destruction of cerebral hemispheres with replacement of the destroyed brain by cerebrospinal fluid [1–7]. Several aetiologies have been postulated for this parenchymal destruction, among which the most often quoted is hypoxic–ischemic changes secondary to a vascular unclear cause affecting the two internal carotids during the second trimester of pregnancy [1–9]. The presented case underwent a medico-legal autopsy together with postmortem enhanced and unenhanced imaging techniques (computed tomography (CT) and magnetic resonance imaging (MRI)).

## Case history

A 21-year-old woman with a denial of pregnancy, delivered at the hospital a male preterm newborn by vaginal birth, during the supposed 22nd and 24th week of pregnancy. There was never any follow-up of this pregnancy and the mother was admitted at the hospital for abdominal pain prior to delivery. The newborn died 4 h after birth and the prosecutor requested medico-legal investigations to determine the cause of death and exclude the intervention of a third person in the death.

One day after death, the body of the newborn underwent an external examination that revealed neither traumatic nor malformative lesions. He weighed 735 g and was 32 cm in length. The anatomy of the umbilical vessels was normal and consisted of two arteries and one vein.

Postmortem imaging investigations prior to medico-legal autopsy consisted of CT and MRI, without and with contrast agent. Unenhanced postmortem CT (PMCT) scans were performed with a LightSpeed VCT 64 from General Electric (Memphis, TN, USA). The scan was performed in four sets: the first set included the brain parenchyma, the second set included the head and neck, the third set included the thorax, the abdomen and the four limbs, and the fourth set included the total body. The first set used the following technical parameters: scan type, helical; detector configuration, 32 × 1.25 mm; tube voltage, 120 kV and 140 mA; rotation time, 1 s; scan field of view (SFOV), ped head, reconstruction algorithms in standard and bone plus. The second set used the following technical parameters: scan type, helical; slice thickness, 0.625 mm; interval, 0.3 mm; tube voltage, 120 kV and 121 mA; rotation time, 1 s; SFOV, ped head; pitch, 0.984:1; adaptive statistical iterative reconstruction (ASIR), 50%; reconstruction algorithms, standard, lung and bone plus. The third set used the following parameters: scan type, helical; slice thickness, 0.625 mm; interval, 0.3 mm; tube voltage, 120 kV and 121 mA; rotation time, 1 s; SFOV, large body; pitch, 0.984:1; ASIR, 40%; reconstruction algorithms, standard, lung and bone plus. The fourth set used the following parameters: scan type, helical; slice thickness, 0.625 mm; interval, 0.4 mm; tube voltage, 120 kV and 121 mA; rotation time, 1 s; SFOV, large body; pitch, 0984:1; ASIR, 60%; reconstruction algorithms, standard.

Unenhanced postmortem magnetic resonance (PMMR) images were performed with an Ingenia 1.5 T from Philips (Amsterdam, Netherlands). Three-dimensional (3D) T1W (weighted) and T2W axial acquisitions were performed on the brain, and the rest of the body was explored through 3D T1W and T2W coronal DIXON.

**Figure 1:**
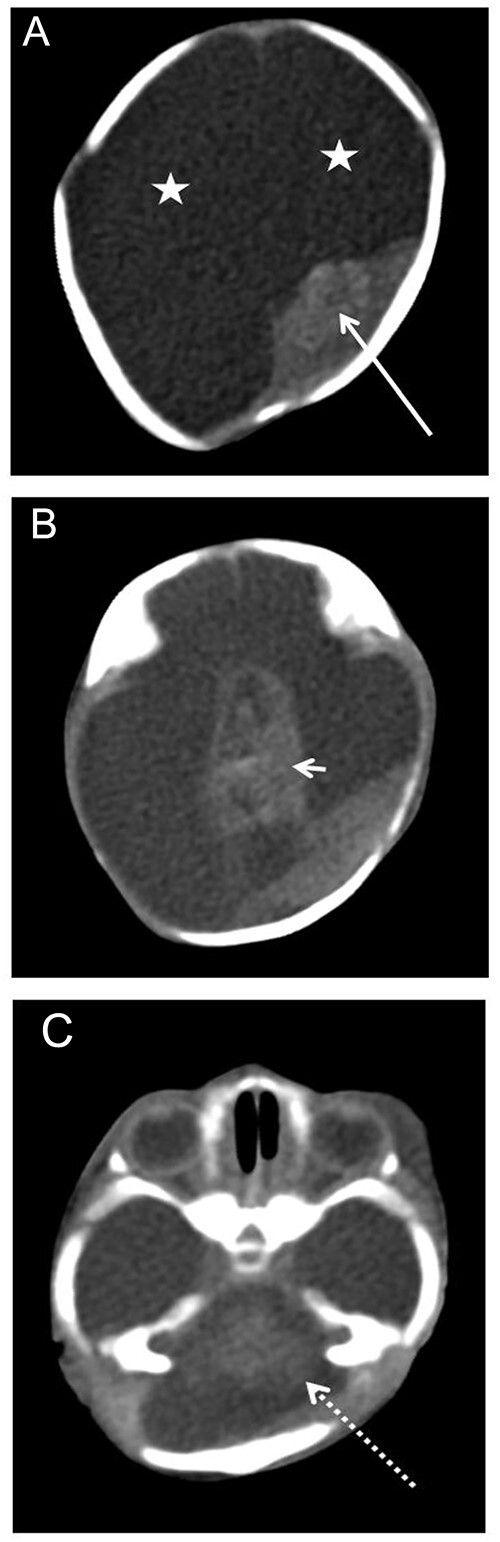
Axial brain postmortem computed tomography (PMCT) images: (A) brain parenchyma replaced by fluid (white stars), an ovoid dense mass instead of posterior part of the left brain parenchyma (long white arrow); (B) hyperdense central mass visible in the supra-chiasmatic region (short white arrow); (C) presence of different cerebellar structures (long dotted white arrow).

**Figure 2:**
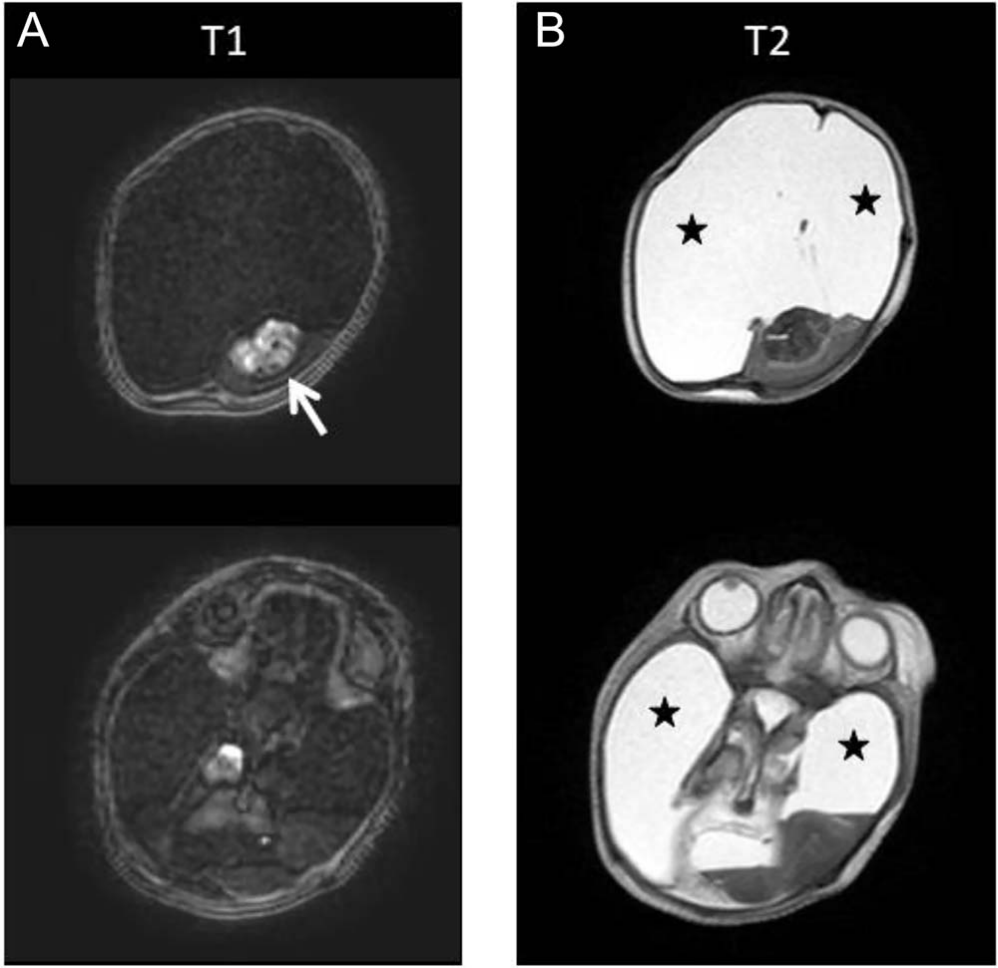
Axial T1W (A) and T2W (B) brain postmortem magnetic resonance (PMMR) confirmed the absence of the cerebral hemispheres and its replacement by fluid (black stars). The central mass was hyperintense on T1W images, suggesting a thalamic mass (white arrow).

**Figure 3:**
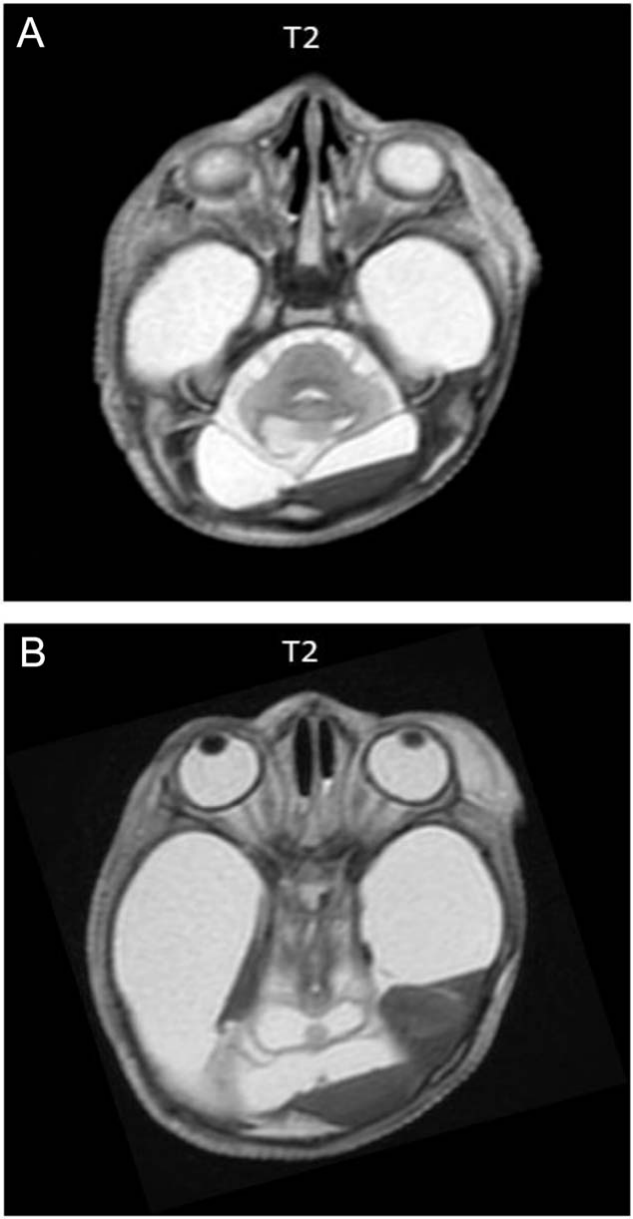
Axial T2W (B) brain postmortem magnetic resonance (PMMR) show the presence of brainstem (A) and both cerebellar hemispheres with cystic changes (B).

Postmortem unenhanced imaging investigations were typical of hydranencephaly. Indeed, PMCT revealed an absence of cerebral hemispheres replaced by fluid, and a hyperdense central mass in the supra-chiasmatic region (Figure 1). Instead of the posterior part of the left brain parenchyma, an ovoid dense mass was visible, surrounded by a hyperdense fluid–fluid level. The brainstem seemed normal. The cerebellar hemispheres presented cystic lesions. No skeletal malformation was noticed on the PMCT. The cerebral PMMR confirmed absence of the cerebral hemispheres replaced by fluid (Figures 2 and 3). The central mass was hyperintense on T1W and corresponded to a thalamic mass. The posterior fluid–fluid level was hyperintense on T1W, as the left posterior mass.

**Figure 4:**
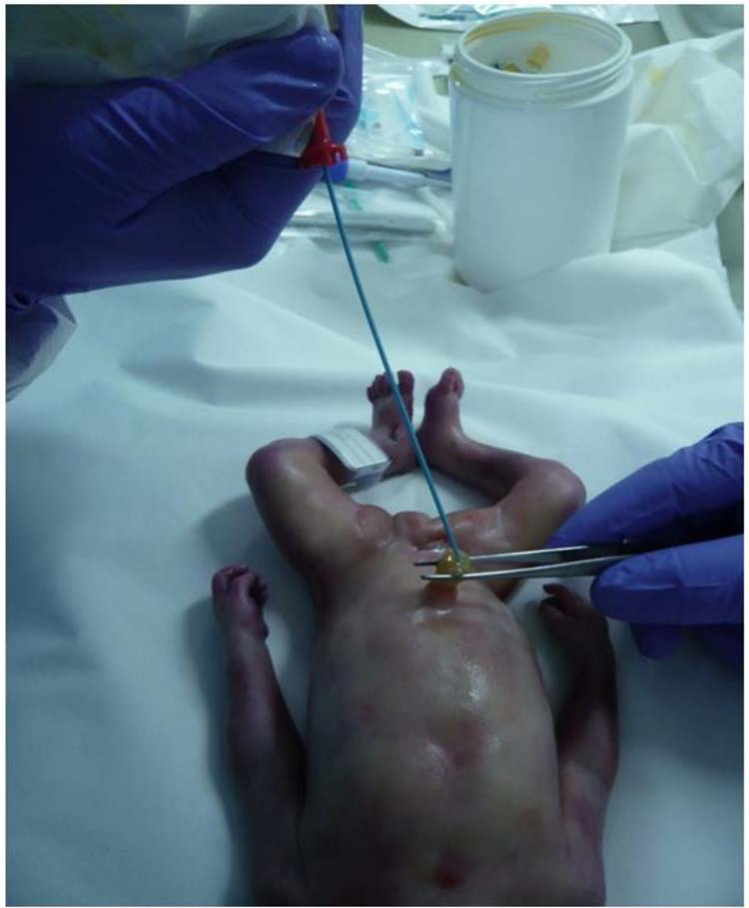
Catheterization of the umbilical vein with a radiological sheath (5 French, Radiofocus Introducer II; Terumo Europe, Leuven, Belgium) prior to angiography.

Then, angiography was performed according to an adaptation of the protocol described by Woźniak et al. [10]. After catheterization of the umbilical vein (Figure 4) and of one of the two umbilical arteries with a radiological sheath (5 French, Radiofocus Introducer II; Terumo Europe, Leuven, Belgium), the oily contrast agent Angiofil® (Fumedica AG, Muri, Switzerland) mixed with paraffin oil (6% dilution) was manually injected in the umbilical vessels (20 mL in the umbilical artery and 20 mL in the umbilical vein) in order to be able to visualize the vascular system of the newborn. A first acquisition was performed after filling of the umbilical artery, and afterwards, we filled one umbilical vein and performed a second acquisition. The two acquisitions used the following technical parameters: scan type, helical; slice thickness, 0.625 mm; interval, 0.3 mm; tube voltage, 120 kV and 121 mA; rotation time, 1 s; SFOV, large body; pitch, 0984:1; ASIR 40%; reconstruction algorithms, standard, lung and bone plus.

PMMR imaging angiography (PMMRA) was performed with a 3D T1W total body acquisition.

**Figure 5:**
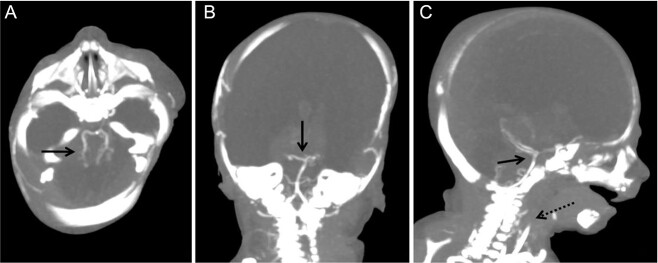
Axial (A), coronal (B), and sagittal (C) brain postmortem computed tomography angiography (PMCTA) (maximum intensity projection, reconstructions) showing a full opacification of the vertebral arteries and basilar trunk with both posterior inferior cerebellar arteries (black arrows), and the absence of opacification of the cervical terminal part of both internal carotids (black spotted arrow). Anterior and middle cerebral arteries are not visible, as well as intra-cerebral veins.

**Figure 6:**
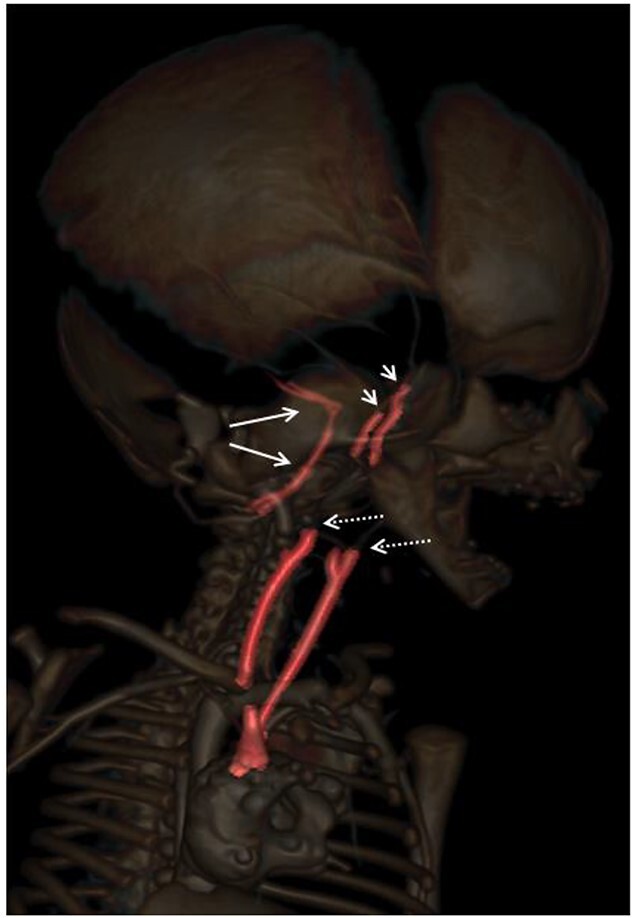
Whole body postmortem computed tomography angiography (PMCTA) (volume rendering, VR) lateral view of the head and antero-lateral view of the neck and superior part of the thorax, showing a full opacification of the vertebral arteries and basilar trunk with both posterior inferior cerebellar arteries (long white arrows), and the absence of opacification of the cervical terminal part of both internal carotid arteries (white spotted arrows). Intra-cranial carotid arteries, in their cavernous portions are partially visible/opacified (short white arrows).

**Figure 7:**
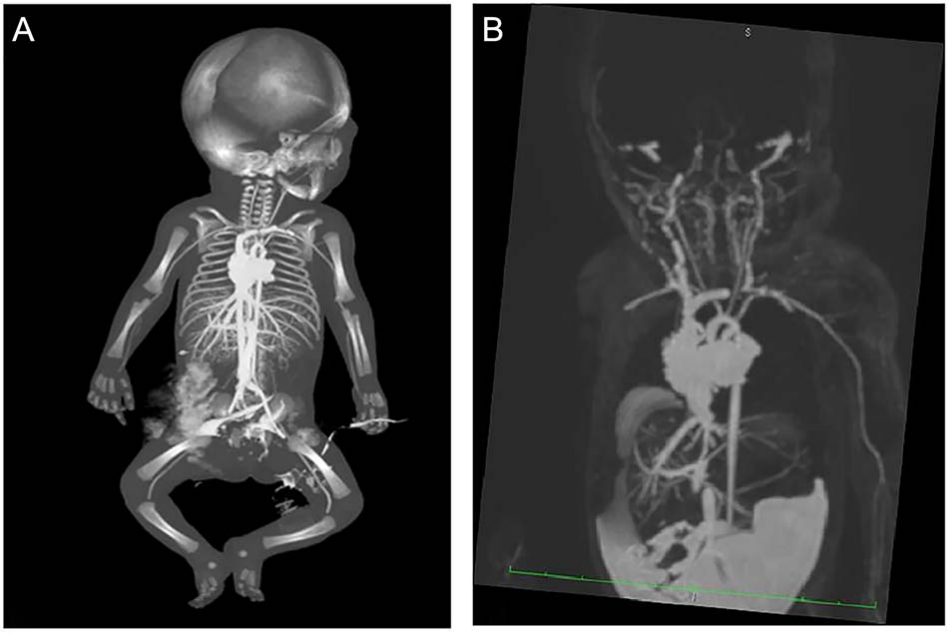
Coronal whole body postmortem computed tomography angiography (PMMRA) (maximum intensity projection reconstructions), general view (A) and zoom (B) showing the absence of signal enhancement of the cervical terminal part of both internal carotids.

PMCT angiography (PMCTA) showed absence of opacification of intracerebral veins. Vertebral arteries and basilar trunk were fully opacified (Figures 5 and 6). The posterior cerebral arteries were proximally visible. Internal carotids, in their cavernous portions were visible, opacified through the posterior communicating arteries. Anterior or middle cerebral arteries were not visible. Cervical terminal part of both internal carotids was not opacified. PMMRA showed a correct filling of venous transverse sinuses and internal jugular veins. Cervical terminal part of both internal carotids was not opacified (Figure 7). Concerning the arterial and venous vascular system of the rest of the body, no other malformation was revealed by the contrast medium.

**Figure 8:**
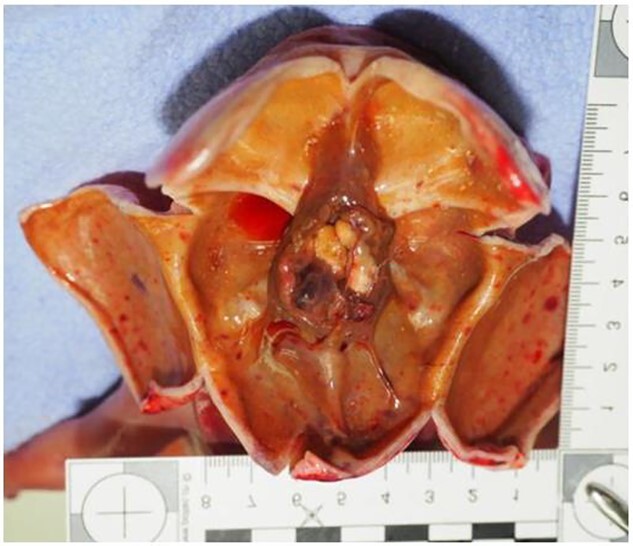
Macroscopic examination of the head. The image shows the opening of the skull with the absence of brain parenchyma.

Finally, a medico-legal autopsy was performed by two board-certified forensic pathologists, and the macroscopic and microscopic examination of the central nervous system was performed by a board-certified neuropathologist. The results of the autopsy, including extensive histological examination of the organs, and of the neuropathological examination (macroscopic and microscopic) confirmed the macroscopic aspect of foetal brain (Figure 8), with parenchymal destruction due to hypoxo-ischemic changes, all corresponding to the histological diagnosis of a massive necrotic-haemorrhagic hydranencephaly. Given the natural cause of death of the foetus and the fact that the intervention or abstention of a third person in the death was excluded, the prosecutor closed the file.

## Discussion

Hydranencephaly is a typical prenatal ultrasound finding, though foetal MRI enables a more precise diagnosis [1, 6, 11]. Characteristic imaging features of classical hydranencephaly are a near-complete absence of the cerebrum supplied by anterior and middle cerebral arteries, hypoplasia of the supra-clinoid internal carotid arteries with normal external carotid arteries, a preserved appearance of basal portions of the temporal and occipital lobes, thalamus, cerebellum, brainstem, and falx, and a typically normal-sized or mildly enlarged cranial vault [1, 2, 7]. The differential diagnosis must be made with hydrocephalus and alobar holoprosencephaly [1, 2].

Hydranencephaly is in first hypothesis believed to be consecutive to an *in utero* vascular impairment regarding the internal carotid arteries, resulting in liquefaction and resorption of the brain parenchyma [1–9]. The development of animal models seems to support this hypothesis [12–14], as well as CT and MRI angiographies of newborns with hydranencephaly [4, 6, 15].

The presented case underwent extended postmortem radiological examinations (PMCT, PMMR, PMCTA, and PMMRA) prior to classical medico-legal autopsy. Indeed, according to the protocol of our institute, an unenhanced PMCT is performed for all autopsy cases. But the visualization of soft tissues is a major limitation of PMCT, which can be partially overcome with PMCTA [16, 17]. However, PMMR has a much better inter-tissular contrast and is a more interesting tool to increase the postmortem diagnostic possibilities. And in this case, PMMRA with the oily contrast agent Angiofil® showed similar results to any clinical MRI contrast agent.

Our case is, to the best of our knowledge, the first case describing the forensic management of a preterm newborn with hydranencephaly including PMCT, PMMR, PMCTA, and PMMRA, which allowed a complete neuro-vascular analysis.

## Authors’ contributions

Coraline Egger collected the data of the study and drafted the manuscript; Bettina Schrag and Tony Fracasso contributed to the medico-legal aspect; Sylviane Hanquinet and Fabrice Dédouit contributed to the radiological aspect. All authors contributed to the final text and approved it.

## Compliance with ethical standards

The study complies with current ethical consideration. The study complies with current ethical consideration and is published with institutional approval from University Center of Legal Medicine Lausanne.

## Disclosure statement

The authors declared no potential conflicts of interest with respect to the research, authorship, and/or publication of this article.

## Funding

This study did not receive any specific grants, funds, or other support for the research, authorship, and/or publication of this article.
